# Hyperreflexic Guillain-Barré syndrome

**DOI:** 10.4103/0972-2327.74195

**Published:** 2010

**Authors:** Neeraj N. Baheti, Davis Manuel, Pranav D. Shinde, Ashalatha Radhakrishnan, Muraleedharan Nair

**Affiliations:** Department of Neurology, Sree Chitra Tirunal Institute for Medical Sciences and Technology, Trivandrum, Kerala, India

**Keywords:** AMAN, autoimmune polyradiculoneuropathy, Guillain-Barré syndrome

## Abstract

Guillain-Barré syndrome (GBS) is an acquired acute autoimmune polyradiculoneuropathy. The 2 features considered essential for the diagnosis of GBS are progressive motor weakness and areflexia. There have been several descriptions of reflex preservation and hyperreflexia in axonal variant of GBS in Chinese, Japanese, and European populations but it is not common in the Indian subcontinent. We report 2 such cases discussing the pathophysiology and management aspects. This case report is to impress upon treating physicians and neurologists in training that a hyperreflexic variant of GBS albeit rare, should not be missed in a given clinical setting.

## Introduction

Guillain-Barré syndrome (GBS) is an acute autoimmune polyradiculoneuropathy, presenting as areflexic, flaccid paralysis with variable sensory disturbances, and elevated cerebrospinal fluid (CSF) protein without pleocytosis.[1] The 2 features essential for a diagnosis of GBS are progressive motor weakness and areflexia. There are 2 distinctive pathologic subtypes of GBS: demyelinating and axonal. Recently, there have been several descriptions of reflex preservation and hyperreflexia in axonal GBS in Chinese, Japanese, and European populations.[1,2] Although this variant is not common in the Indian subcontinent, a few cases have been reported.[3,4] A high index of suspicion is needed to diagnose this rare presentation of GBS. We report 2 such cases discussing the pathophysiology and management aspects.

## Case Reports

### Case 1

A 50-year-old male was admitted with fever with abdominal pain and diarrhea lasting 2 days, which improved with symptomatic treatment. Over the next 4 days, he had progressive weakness of limbs and he needed 1 person’s support to walk. There were no cranial nerve, sensory, or bladder symptoms. There was no muscle pain. There was no other antecedent illness or recent vaccination. On examination, limbs were flaccid, and power of the proximal muscles of the lower limb was 3/5 (Medical Research Council grading) and distal muscles was 2/5. In the distal upper limb muscles, power was 3/5. There was mild truncal weakness. Deep tendon reflexes were brisk throughout the course of illness and plantars were flexor bilaterally. Sensory and cerebellar system was normal. Investigations showed normal total and differential leukocyte counts, erythrocyte sedimentation rate (9 mm/h), and creatine kinase and electrolytes (Na, Ca, K, Mg, phosphate). Vasculitis work-up was negative. CSF examination showed 2 cells (100% lymphocytes). CSF sugar was 74 mg/dL (plasma glucose 121 mg/dL) and protein was 68 mg/dL. Magnetic resonance imaging of brain and spine were normal. Nerve conduction study [Table 1] was suggestive of pure motor axonopathic variant of GBS (acute motor axonal neuropathy [AMAN]). He was treated with intravenous immunoglobulin (IVIg) (400 mg/kg/day) for 5 days and he improved. At discharge 2 weeks later, he had a significant improvement and upper limb power was normal and lower limb power was 4/5 proximally and 3/5 distally.

**Table 1:** Table showing nerve conduction parameters of the 2 patients

Case 1 (Day 4 of illness)	Median N		Ulnar N		Tibial N		Peroneal N		Sural N	
	Rt	Lt	Rt	Lt	Rt	Lt	Rt	Lt	Rt	Lt
CMAP latency (ms)	3.0	3.2	2.7	2.7	5.0	4.2	3.9	5.0	–	–
CMAP amplitude (mV)	5.1	6.6	3.0*	5.8	1.2*	0.6*	1.1*	0.7*	–	–
MCV (m/s)	59.0	59.0	63.6	62.2	45.1	50.6	45.3	51.4	–	–
Sensory onset latency (ms)	2.4	2.3	2.2	1.9	–	–	–	–	1.9	2.2
Sensory peak latency (ms)	3.1	2.9	2.8	2.5	–	–	–	–	2.6	2.8
SNAP amplitude (µV)	38.2	42.7	25.2	25.2	–	–	–	–	11.2	12.2
F-latency	26.1	26.7	NR*	NR*	NR*	NR*	NR*	NR*	–	–

**Case 2 (Day 6 of illness)**										

CMAP latency (ms)	3.7	3.2	2.4	2.2	4.8	4.9	4.7	4.4	–	–
CMAP amplitude (mV)	11.0	15.0	7.0	3.5*	1.5*	1.0*	0.8*	0.5*	–	–
MCV (m/s)	58.8	58.8	64.7	59.4	40.2	47.8	53.5	50.8	–	–
Sensory onset latency (ms)	2.2	2.0	1.9	1.8	–	–	–	–	2.8	3.0
Sensory peak latency (ms)	2.8	2.6	2.4	2.3	–	–	–	–	3.4	3.6
SNAP amplitude (µV)	65.2	76.7	53.0	61.6	–	–	–	–	17.3	17.7
F-latency	24.7	23.1	24.3	NR*	NR*	NR*	NR*	NR*	-	-

Rt, right; Lt, left; CMAP, compound muscle action potential; MCV, motor conduction velocity; SNAP, sensory nerve action potential; NR, no response.

* Abnormal values

### Case 2

An 18-year-old female was admitted with progressive weakness of limbs of 6 days duration. She had diffuse muscle aches. She could barely walk few steps with 2 persons’ support. There were no cranial nerve, sensory, or bladder symptoms. She had fever, abdominal pain, and diarrhea 4 days prior to weakness, lasting 2 days, which improved with symptomatic treatment. No other antecedent illness or vaccination was reported. On examination, limbs were flaccid, and power of proximal muscles of lower limb was 3/5 and distal muscles was 1/5. The power of proximal and distal muscles of the upper limb was 4/5 and 3/5, respectively. Deep tendon reflexes, including pectorals and trapezius, were brisk throughout the course of illness and plantars were flexor bilaterally. Sensory and cerebellar system was normal. All hematologic work-up, including creatine kinase was normal. CSF sugar was 64 mg/dL (corresponding plasma glucose 98 mg/dL) and protein was 60 mg/dL. Magnetic resonance imaging of brain and spine were normal. Nerve conduction study [Table 1] showed features suggestive of acute pure motor axonopathic variant of GBS. She was treated with large volume plasma exchange with significant improvement at 2 weeks with only mild dorsiflexor weakness.

We could not do anti-*Campylobacter jejuni* antibodies, anti-GM1, GM1b, and GD1a ganglioside antibodies in both the cases due to local unavailability.

## Discussion

GBS is a group of syndromes with several distinctive subtypes classified on a pathologic basis into demyelinating and axonal forms. Axonal GBS has been classified further into 2 groups: AMAN and acute motor and sensory axonal neuropathy.[3] Although hyporeflexia or areflexia is the hallmark of GBS, normal reflexes or hyperreflexia is not a finding inconsistent with the diagnosis of GBS. The variants most commonly reported to be associated with retained or brisk reflexes are AMAN, acute motor conduction block neuropathy, and acute facial diplegia with brisk reflexes.[1,5,6] The incidence of hyperreflexia in AMAN is reported to be between 33% and 48%.[1,2]

Hyperreflexia is seen in GBS associated with antecedent *C. jejuni* infection; most of them have history of abdominal pain and diarrhea. These cases are usually mild and significant bulbar or respiratory involvement is uncommon. CSF analysis shows albuminocytologic dissociation in most cases. Almost all of them have IgG anti-GM1 ganglioside antibodies although anti-*C. jejuni* antibodies are frequently negative.[1] Antibody testing is not freely available in developing countries, such as India, which makes the diagnosis more difficult.

Although preservation of reflexes may simply be due to sparing of the sensory afferent pathway, the occurrence of brisk reflexes suggests a central mechanism. Dysfunction of inhibitory systems in the spinal interneurons has been proposed.[1] In these cases, distal conduction disturbance, not axonal degeneration, produces low motor responses on nerve conduction studies, termed as reversible conduction failure or acute motor conduction block neuropathy.[2] The presumed mechanism producing reversible conduction block is impaired physiologic conduction at the nodes of Ranvier. One limitation of the study is that needle electromyography (EMG) examination was not done for both the cases. It would have strengthened the above hypothesis. None of the previous reports till date have done EMG in the above scenario.[1,4–6] Although in general, AMAN has been associated with extensive axonal loss and poor outcome, this subgroup with reversible conduction failure recovers rapidly.[1,2,6] The response to both IVIg and large volume plasma exchange is similar. Both our cases also improved significantly at the end of 2 weeks.

The most common differential diagnosis presenting in a similar manner with acute progressive weakness and brisk reflexes is a high cervical myelopathy. Eliciting a careful history and a preceding history of enteritis, with electrophysiologic and CSF examination makes the diagnosis clear.

Therefore, GBS (axonal form) should be considered in patients with acute pure motor quadriparesis with normal or brisk reflexes, especially with antecedent gastroenteritis. This case report is to impress upon treating physicians and neurologists in training that a normo/hyperreflexic variant of GBS albeit rare, should not be missed in a given clinical setting.
